# “I want to know everything”: a qualitative study of perspectives from patients with chronic diseases on sharing health information during hospitalization

**DOI:** 10.1186/s12913-017-2487-6

**Published:** 2017-08-04

**Authors:** Marge Benham-Hutchins, Nancy Staggers, Michael Mackert, Alisha H. Johnson, Dave deBronkart

**Affiliations:** 1 School of Nursing, University of Texas at Austin, 1710 Red River (D0100), Austin, TX 78701 USA; 2 Department of Biomedical Informatics and College of Nursing, University of Utah, 5977 E. Pioneer Fork Road, Salt Lake City, UT 84108 USA; 3 Department of Population Health, Stan Richards School of Advertising & Public Relations, University of Texas at Austin, 300 W. Dean Keeton (A1200), Austin, TX 78712 USA; 4 Society for Participatory Medicine, 17 Grasmere Lane, Nashua, NH 03063 USA

**Keywords:** Self-care, Self-management, Health literacy, Patients, Chronic disease, Patient participation, Qualitative research, Hospitalization, Nursing, Communication

## Abstract

**Background:**

Patient-centered care promotes the inclusion of the most prominent and important member of the health care team, the patient, as an active participant in information exchange and decision making. Patient self-management of a chronic disease requires the patient to bridge the gap between multiple care settings and providers. Hospitalizations often disrupt established self-management routines. Access to medical information during hospitalization reflects patients’ rights to partner in their own care and has the potential to improve self-management as well as promote informed decision making during and after hospitalization. The objectives of this study were to elicit the perspectives of patients with chronic disease about desired medical information content and access during hospitalization.

**Methods:**

This exploratory study incorporated a qualitative approach. The online survey included the research team created open and limited response survey, demographic and hospital characteristic questions, and the Patient Activation Measurement instrument (PAM®). Convenience and social media snowball sampling were used to recruit participants through patient support groups, email invitations, listservs, and blogs. The research team employed descriptive statistics and qualitative content analysis techniques.

**Results:**

The study sample (*n* = 34) ranged in age from 20 to 76 (*μ* = 48; *SD* = 16.87), Caucasian (91%, *n* = 31), female (88%, *n* = 30) and very highly educated (64%, *n* = 22 were college graduates). The PAM® survey revealed a highly activated sample. Qualitative analysis of the open-ended question responses resulted in six themes: *Caring for myself*; *I want to know everything*; *Include me during handoff and rounds*; *What I expect*; *You’re not listening*; and *Tracking my health information*.

**Conclusions:**

This study revealed that hospitalized patients want to be included in provider discussions, such as nursing bedside handoff and medical rounds. Only a few participants had smooth transitions from hospital to home. Participants expressed frustration with failures in communication among their providers during and after hospitalization and provider behaviors that interfered with patient provider communication processes. Patients also identified interest in maintaining their own health histories and information but most had to “cobble together” a myriad of methods to keep track of their evolving condition during hospitalization.

**Electronic supplementary material:**

The online version of this article (doi:10.1186/s12913-017-2487-6) contains supplementary material, which is available to authorized users.

## Background

Patient-centered care promotes the inclusion of the most prominent and important member of the health care team, the patient, as an active participant in medical information sharing and decision making [1]. This process acknowledges and builds on patient autonomy. It is important to recognize that patients are not alone in this process; they are guided by knowledgeable health professionals with the focus on empowering, engaging, equipping and enabling patients as members of the health care team [2]. The patient is often faced with bridging the gap during transitions between providers and care settings, including their own home [3, 4]. Despite its importance, few studies have explored patient perspectives about this critical transition. This study intends to help fill this gap by identifying patient perceptions of self-management during hospitalization and the immediate time period after discharge. The objectives of this study were to elicit the perspectives of patients with chronic disease about desired medical information content and access during hospitalization, desire for shared decision making, and how an inpatient hospitalization influences chronic disease self-management.

Hospitalization has been identified as “one of the most disempowering situations” that an individual experiences in our modern society [5]. When patients with a chronic condition are admitted to the hospital they are expected to switch from being the leader of their own care to a passive consumer and upon discharge once again resume self-management. Patient stories of unanswered call bells, autocratic providers, lack of access to the results of tests [2, 6–8], and unavailability of services on weekends [9] reveal a provider-centric inpatient environment. Although patient discharge paperwork provides a summary of the hospitalization and basic instructions, the full medical record, including the results of laboratory and imaging tests during the hospitalization, is not easily obtained by the patient [10]. Clearly the lack of access to inpatient records during hospitalization and difficulties obtaining records after discharge limits the ability of the patient, family, and outpatient providers to manage care optimally after discharge [11, 12].

Patient-centered care promotes the inclusion of the patient as an active participant in information exchange and decision making across the continuum of care [13–19]. Bedside nursing shift change handoff and medical rounds provide excellent opportunities for hospitalized patients and providers to exchange information [20]. Although patient participation is clearly important, the current process and structure of bedside handoff and rounds rarely encourages it [21]. While general checklists and protocols are available to support provider-centered handoffs [22, 23] and rounds [21], no published tools are available to guide patient participation. Patient participation in bedside nursing shift change handoff and medical rounds during hospitalization reflect the patient’s right to partner in their own care and has the potential to improve self-management and promote informed decision making during and after hospitalization [24, 25].

Staggers, Benham-Hutchins, and Langford explored patient participation in bedside handoff and the tools used to keep track of inpatient activities [24, 26]. Patients used ad hoc methods, such as handwritten notes, in-room whiteboards, reliance on family members, and their own memories to keep track of inpatient procedures and test results. Findings concurred with previous studies [27, 28] that revealed patients want to be active participants in their care, when their health status allows it, and that insecurity about their role has the potential to inhibit participation.

Past authors identified the value of active patient involvement in these processes for continuity of providers, and structural barriers, such as the way care was delivered or organized, were barriers to participation [27, 29]. Research has shown that patients were reluctant to speak up about changes in their own condition [29, 30] and, even when invited to participate, patients have concerns about overstepping established social roles, physician authority, and the fear of being labeled “difficult” [31]. This may lead to patients feeling insecure or fearful about expressing views due to the imbalance of power in the provider-patient relationship [32].

Better understanding of this topic is critical to improve patient participation and care compliance, to smooth the transition back to self-care, and in the long term, improve outcomes. In particular, understanding the current gaps in patients’ ability to assume self-care may point to specific methods for improving the process in the near term. To restate, the purpose of this research was to describe patients’ perceptions about self-management during hospitalization and the immediate time period after discharge.

## Methods

### Study design

This exploratory study incorporated a qualitative approach to determine patient identified information needs, how an inpatient hospitalization influences patient self-management of chronic disease across care transitions, patient perceptions of facilitators and barriers to shared decision making during hospitalization and level of participation in shift change bedside handoffs and medical rounds.

### Research team

The research team was made up of individuals with a broad range of experiences, including patient engagement, health literacy, academic research, informatics, methodological experts, nursing practice, informatics, and the unique perspective and expertise of a cancer survivor and patient participation movement leader.

### Human subject protection

University Institutional Review Board (IRB) approval was obtained. Potential participants had the option to access a study web page for additional information or directly access the survey. The survey opened to the IRB approved consent form. Potential participants were instructed that clicking on a clearly labeled button meant that they had read the consent and were agreeing to participate in the study. Participants were not required to answer any specific question and could decide at any time not to continue. At the end of the survey participants were presented with a clearly labeled button to submit their responses.

### Setting and sample

To clearly distinguish this study from specific hospital satisfaction surveys, convenience and social media snowball sampling (SMSS) were used to recruit participants (*n* = 34) through online patient support groups, email invitations, listservs, blogs, and social media. We chose this more novel recruitment approach because SMSS incorporates traditional snowball sampling methods that utilize the social network of participants to identify potential participants, and incorporates the use of online social media, such as Facebook®, to enhance recruitment through the sharing of study information. The online survey was selected for this study to reach potential participants across the US and across urban, suburban, and rural areas. Inclusion criteria were: age 18 and up, living in the United States, diagnosed with a chronic disease, and have had an inpatient hospitalization after initial diagnosis. In addition, participants must have been able to read, write and speak in English and have access to the Internet to complete the online questionnaire.

### Questionnaire

An online survey with limited choice and open-ended questions was developed by our team to support a mixed methods approach and to collect both qualitative and quantitative data. The online questionnaire consisted of three components: (1) demographics, (2) the Patient Activation Measure (PAM®) survey and (3) investigator-developed, limited response and open-ended questions about patients’ hospital experiences. Survey components 1 and 3 are available as Additional file 1. Information on licensing the PAM® survey is available through Insignia Health (http://www.insigniahealth.com/products/pam-survey). Demographics included: age, education, gender, ethnicity, US region, type of chronic disease, and hospital characteristics. Participants completed the PAM® survey that consists of 10 statements about self-management [33] and indicates the level of patient engagement in self-care. The instrument uses a 4-point Likert Type Scale for respondents to indicate agreement or disagreement with each statement. The items form an interval level, unidimensional, Guttman-like scale with strong psychometric properties [34]. The full measure is scored on a scale from 0 to 100, with scores corresponding to patient activation level. Level 1 (0.0–47.0) corresponds to an individual not yet understanding their self-management role; Level 2 (47.1–55.1) indicates the individual may lack knowledge or confidence; Level 3 (55.2–72.4) reveals the beginnings of engagement and Level 4 (72.5–100.0) indicates that the individual engages in self-management behaviors.

Our research team collaborated on the development of limited response and open-ended questions (Table 1) designed to elicit patient viewpoints on existing self-management practices, the influence of hospitalization on self-management, information access during hospitalization, and participation in nursing bedside handoff and/or interdisciplinary rounds. In addition, interview questions from earlier work by two of the research team members (NS and MBH) were modified for this study [24, 26].

**Table 1:** Open ended and limited response questions

• How well was your medical care organized while you were in the hospital?
• How often did your providers seem to know what they needed to know about you, your medical care, and what you needed?
• Please share how you keep track of your health information as you go from provider to provider and setting to setting.
• Please share how you keep track of your health information while you are in the hospital.
• What kinds of medical information are you interested in knowing about when you are in the hospital?
• What medical information do you prefer not to know about when you are in the hospital?
• Did the nurses give a change of shift report in your hospital room? If yes: How often did this occur? Did the nurses explain what they were doing? Were you invited to participate? What did you do during the process? What factors do you think encourage patient participation in bedside nursing shift report? What factors do you think discourage patient participation in bedside nursing shift report?
• During your hospitalization, did your doctors and other providers discuss your medical care as a group (medical rounds) in your hospital room? If yes: How often did medical rounds occur in your hospital room? Did the medical team explain what they were doing? Were you invited to participate? What did you do during the process? What factors do you think encourage patient participation in medical rounds? What factors do you think discourage patient participation in medical rounds?
• When you first got home from the hospital, how confident were you that you knew what you would need to do to take care of your medical needs?
• As time went by (a week or more after discharge), did you find that you actually did know what you needed to do to take care of yourself?
• Did you find that you had the things you needed -- equipment, prescriptions, or anything else?
• Did you find that you did know what to do if problems developed?
• Did you find that you got the help you needed, when you needed it?
• Thinking back on your hospitalization, is there anything else that you think could be done during hospitalization to help prepare you to resume self-care after you went home?

### Data management

All data were collected electronically. Participant identifying information was not collected. The Qualtrics® survey management system, licensed and approved by the university information security office, was used to create and distribute surveys and store survey responses (https://www.qualtrics.com/research-core/). University approved cloud storage supported secure data storage and collaboration between the research team members. All data were anonymously coded for analysis and reporting.

### Data analysis

The research team employed descriptive statistics and qualitative data analysis techniques [35–37]. IBM® SPSS® statistical software was used for quantitative analysis for demographic data and the limited response questions. These data and the PAM® survey items were analyzed using descriptive statistics. The PAM® instrument is scored on a scale from 0 to 100, with scores corresponding to patient activation level, with levels indicating progressively higher levels of patient activation.

Transcripts of the open-ended question responses were checked for accuracy and then imported into Atlas ti™ v7.5.13 qualitative software. The analysis followed the steps of conventional qualitative content analysis [35–37]. Three members of the research team (MBH, NS, MM) independently read each open-ended response and identified key words or phrases to be used as codes to index meaningful segments. Both descriptives and theoretical codes were employed as the open-ended responses were read, interpreted, and discussed with the rest of the research team. Next the team moved to the second step: an iterative process of individual coding of all of the transcripts and review of the transcripts for coding consistency. The investigators jointly compared individual analyses, reconciled any differences of perspective, and achieved consensus on the codes and their application. The third step involved consolidation of categories and theme identification, using an inductive process. To maintain rigor throughout the analysis, features of trustworthiness were given careful attention: confirmability to maintain neutrality and remain true to the participants’ views; auditability as it pertains to the process of inquiry, findings, interpretations, and recommendations; and credibility via member checks [38].

## Results

Thirty-four participants submitted completed surveys. Participants ranged in age from 20 to 76 (*μ* = 48; *SD* = 16.87). The majority of respondents were Caucasian (91%, *n* = 31) and female (88%, *n* = 30). The sample was very highly educated, with 29% (*n* = 10) having some college or technical school, 32% (*n* = 11) were college graduates and 32% (*n* = 11) had some graduate school or an advanced degree. The remaining participants (*n* = 2) indicated they were high school graduates. Hospitals in all four geographical regions of the United States as well as urban, rural, and suburban settings were represented (Table 2). The results of the PAM® survey revealed a highly activated sample: Level 1 (0); Level 2 (3); Level 3 (21) and Level 4 (10).

**Table 2:** Hospital setting and region

		United States Regions				
		Northeast	Midwest	South	West	Total
Setting	Urban	4	5	10	3	22
	Rural	0	0	1	0	1
	Suburban	1	1	6	2	10
	Total	5	6	17	5	33

Recruitment began with a focus on cancer, as a primary chronic disease, and then was expanded to include people with other chronic diseases. Consequently, the study sample (*n* = 34) includes more participants with cancer (15) than other chronic diseases: Type 1 Diabetes (3); Gastrointestinal (6); Lupus (2); Pulmonary (2); Musculoskeletal (3); Integumentary (2) and Hematologic (1).

We asked participants about patient/provider communication tools and activities. Twenty-four participants (71%) indicated that they had a whiteboard in their hospital room. Only 10 participants (29%) recalled having nursing shift change handoff report in their hospital room with four reporting that they were usually or consistently invited to participate. When asked what they did during the handoff report all ten indicated they listened, four asked questions, five answered questions, and two filled in the “other” response with “make corrections.” Fifteen participants (44%) indicated that medical rounds occurred in their room with only two individuals indicating they usually or consistently were invited to participate. This group indicated they listened (13), asked (11) or answered (11) questions during rounds.

We also asked participants (*n* = 34) whether, as time went by (a week or more after hospital discharge), they felt prepared to care for themselves at home. Most (76%) indicated they felt prepared but a substantial percent (21%) indicated that they did not know what they needed to know to take care of themselves at home. When asked about the availability of specific resources 11.8% (4) indicated they did not have the equipment or prescriptions they needed. In addition, 5.9% (2) indicated they did not know what to do if problems developed and 8.8% (3) did not know who to contact with questions or problems.

## Themes

The qualitative analysis of the open-ended question responses resulted in 533 codes synthesized into 16 categories and six themes (Table 3). Participant gender, age, and study ID number are provided in brackets after quotes.

**Table 3:** Themes and categories with code frequencies

Themes and categories	Frequencies	
	Theme	Code
Caring for myself	148	
Care coordination		38
Discharge information		22
Provider contact post discharge		29
Self-Care		59
I want to know everything	97	
Communication - inpatient		25
Here is the information I want		72
Include me during handoffs and rounds	49	
Bedside shift report: barriers and facilitators		19
Medical rounds: barriers and facilitators		30
What I expect	83	
Communication - interprofessional		23
Patient expectations: care process		26
Patient perception of provider knowledge and recommendation		34
You’re not listening	87	
Communication - patient-provider		53
Invisible patient		17
Listen to the patient		17
Tracking my health information	69	
Information tracking: inpatient		28
Information tracking: outpatient		41
Total	533	

### Caring for myself

The focus of this theme is participant views and experiences with resuming self-management after discharge from the hospital. Specifically, participants were asked about how prepared they were when they first got home from the hospital and a week or more after discharge. This theme integrates four categories: *care coordination*, *discharge information*, *provider contact after discharge* and *self-care*.

Seven participants stated they had acceptable discharge instructions, including provider contact information for questions or problems. The remainder identified problems that interfered with provider/patient care coordination and self-management. Problems included: poor referrals and issues identifying the right provider for follow up; medication related issues including identification of the right provider to follow up with hospital prescribed medication; only being told to go to the emergency department if they had problems, lack of knowledge about their disease prognosis, and knowledge about the supplies needed at home.

Multiple participants identified that they experienced a total lack of post discharge care coordination, “I had no idea who or which doctor to call” [F, 48,114] and “I developed blisters from a medication side effect and did not know what to do” [F, 21,131]. Another participant described their discharge process, “…never advised in-person of a follow-up/survivor plan – simply handed a document saying I would be contacted later and a prescription” [F, 68, 119]. Summing up the problem, one oncology patient stated, “Generally speaking, the oncology medical community is not addressing the needs of those of us living with lifelong treatment and chronic cancers” [F, 54, 109].

Last, patients noted the importance of individual patient involvement in their own care and family or caregiver advocacy with one participant stating, “I was alone with no family or friends available to act as advocates” [F, 68, 119]. Participants also mentioned patient support communities and internet searches as resources they used and were readily available for self-management information after discharge. One patient stated, “I am generally able to get specific, immediately applicable advice from my online patient community and PubMed or other trusted online sources. It would be helpful for discharge instructions to make note of these resources and their pros and cons [F, 37, 111].

### I want to know everything

This code incorporates two categories: *communication - inpatient* and *here is the information I want*. Specifically, participants were asked what medical information they would like to know about while in the hospital and what medical information they would prefer not to know. Participants overwhelmingly indicated that they wanted to know everything, although one participant preferred not to know about the prognosis: “I don’t need to know long term complications and morbidity related to my condition” [F, 64, 157].

Four participants specifically mentioned concern about the possibility of providers withholding information: “I want nothing of my own concealed from me” [M, 56, 121]; “I’d rather be over-informed than under. Don’t keep information from me. I need to feel like I can trust you and your judgement. Do not sugar coat things” [F, 28, 136]; “I want my health care team to be up front with me” [F, 32, 138]; and “I’d prefer to know everything, and I’d actually prefer them not to hide anything from me” [F, 22, 158]. Specific desired information identified by the participants include: surgery/treatment/pathology results – including copies and explanations before discharge, treatment and medication information and side effects, the information necessary to be involved in the decision-making process, and post discharge self-management skills.

Participants also voiced their desire for education and information while hospitalized to help them plan for discharge. This included a desire to know “what to expect and what milestones I needed to make before I would be allowed to go home” [F, 56, 113] and their “expected health status after discharge and the type of follow up care required’ [F, 68, 119]. One participant stated a desire to know about “the projected long-term course of treatment… resources to support the cost of treatment” and “community resources for connecting with other cancer patients for managing the impact of the disease and treatment” [F, 54, 109].

### Include me during handoffs and rounds

This theme integrates two categories: *bedside shift report-barriers and facilitators* and *medical rounds-barriers and facilitators*. Participants were asked to identify factors that encourage/discourage patient participation in bedside nursing handoff and medical rounds. One patient poignantly summed up their experience, “My hospital is a teaching hospital, so my care team came with an army of medical students, and discussions about my situation and my care were had while I was sitting in the room with them, only they did not actively acknowledge my presence, so I felt like an animal at the zoo” [F, 37, 133]. Another participant expressed a “sense of powerlessness and intimidation” [F, 68, 119] during rounds. Patient condition was also identified as a potential barrier to participating: “pain meds cause a foggy brain and difficulty concentrating” [F, 35, 135]. In addition, participants identified numerous provider behaviors that discouraged patient participation (Table 4).

**Table 4:** Provider behaviors that discourage patient participation

“…talking around the computer in hushed tones, not directly addressing the patient, lack of eye contact.” [F, 20, 108]	
“…disinterest and lack of time.” [F, 56, 113]	
“Speaking as if the patient is not there, using jargon and acronyms, condescension of any sort.” [F, 37, 111]	
“Arrogance. Seeming to be in a hurry and believing they know what you feel.” [F, 67, 120]	
“Treating the patient as a number and not a person.” [F, 37, 133]	
“…too many people in the patient’s room.” [F, 35, 135]	

Factors that encourage patient participation include acknowledging the patient, inviting them to participate and to “make sure the patient is introduced to all the players and made aware of why they are participating and their contribution to the patient’s care” [F, 68, 119]. Two participants articulated the importance of the patient feeling as if they are part of the process: “patients need to be aware of the importance of their contribution” [M, 76, 118] and “Doctors, etc., should talk to/with the patient, not just about the patient. They should encourage the patient to ask questions, give them permission, in a sense, to be part of the discussion” [F, 64, 157].

### What I expect

The focus of this theme was hospitalized patients’ expectations about communication, care process and provider knowledge. This theme was derived from three categories:

*Communication-interprofessional*, *patient expectations-care process*, and *patient perception of provider knowledge and recommendations*. Patients were specifically asked to comment on how their care was organized while they were in the hospital and if their providers seemed to know what they needed to know about them, their medical care and what they needed. Coordination of care and evidence of inter-professional communication were common patient expectations. In contrast, two patients shared concerns about communication between nursing shifts and frustration at “having to go over the same info with each shift change” [F, 37, 111] and being faced with the need to “…give my spiel all over again” [F, 38, 134] every time they had a new nurse. In addition, two patients questioned the physician’s knowledge of the patient’s specific case: “doctors needed to read the file” [F, 29, 129] and “…the two hospitalists asked questions that made it seem like they hadn’t listened at all or read my records” [F, 41, 154].

Although one participant stated: “my care is beautifully coordinated between my medical team…they anticipated everything” [F, 67, 120] other participants voiced concerns about inter-professional collaboration. One participant stated that the physicians collaborated well with each other, “but the nurses were left out of the treatment plan loop” [F, 35, 135]. Concern about “redundant testing, poor inter-professional collaboration, and specialists’ recommendations [being] disregarded” [F, 21, 170] was shared by another participant. In addition, participants expressed the desire to be involved in their own care: “I’d like to be part of the decision process” [F, 37, 133] and for providers to “acknowledge the patient and respect their perspectives and opinions’ [F, 37, 133].

### You’re not listening

This theme incorporates three categories: *communication (patient-provider)*, *invisible patient*, and *listen to the patient*. This theme emerged in response to questions about provider knowledge and coordination of care. Participants reported providers ignoring or dismissing their questions and treatment or medication preferences, to the point of “discounting things I’ve told them on the spot” [F, 37, 111]. One patient was given an intravenous (IV) medication against her “stated wishes” [F, 63, 103]. This resulted in an allergic reaction and the participant angrily stating, “LISTEN TO THE FREAKIN’ PATIENT, folks” [F, 63, 103].

Dismissing the patient’s point of view (“I never got the idea they wanted my opinion” [F, U, 126]) and redirecting or partially answering questions (“What I wanted was more thorough information, and to not have what I was asking disregarded” [F, 21, 170]) were also identified as leading to patients feeling ignored and invisible. Contrasting experiences with providers acknowledging patient reported pain were shared: “No one would acknowledge [my] pain, nerve pain that developed early on and continued” [F, 68, 119] and “My medical team listened to me when I said that I was in pain, which is huge because I’ve had others dismiss the pain I’m in” [F, 22, 158].

### Tracking my health information

This theme incorporates two categories: *outpatient information tracking* and *inpatient information tracking.* Participants were asked how they keep track of their health information between outpatient providers and settings and, specifically, how they kept track while hospitalized. Responses revealed that patients use multiple methods to keep track of their health information both in and out of the hospital. Seven (12%) of participants used manual methods, four indicated they only used paper records, one relied on his own memory, and two stated that they depend on family members to keep track for them. Paper records consisted of calendars, journals, logs, and paper file folders containing printouts of medical records.

Twenty (59%) indicated that they used both paper and electronic methods and eight (24%) identified only electronic methods. Electronic methods included multiple patient portals, digital notes, computer applications, and smart phone apps (including taking photos of paper records). Some patients created their own method to pull together and summarize the records from multiple sources to share with providers. One participant described creating “a cheat sheet to carry with me with a quick reference to all surgeries, problems, medications, allergies, etc., and is updated as changes occur” [F, 67, 105] and another developed a “consolidated, ready-reference document” [F, 68, 119] that contained information from provider records, a timeline of events, and his own notes.

## Discussion

Our findings build on previous studies identifying that hospitalized patients with chronic conditions are faced with challenges as individual as their personal circumstances, goals and priorities [4, 13, 18, 24, 26]. Only a few participants had smooth transitions from hospital to home. Poor patient-provider communication [27, 31] and lack of access to the electronic health record during hospitalization [7, 8, 12] were shown to interfere with the development of self-management skills. Many participants in this study identified significant communication problems during hospitalization that created a sense of insecurity and hindered their ability to resume self-management after discharge. Inconsistent discharge practices and communications regarding readiness for discharge also led to feelings of anxiety and uncertainty.

Data indicated many patients want to know everything, but current methods of sharing patient information in a hospital setting often inhibit patient participation. Many participants expressed frustration with failures in communication among their providers during and after hospitalization. This suggests an ongoing need for inter-professional training and improved communication among teams to alleviate such breakdowns in coordination and communication. Better systems and structures for communication and training around inter-professional coordination and communication that includes patients are clearly needed [12, 19, 39].

Poor communication has been identified as a major factor leading to medical error [17, 20]. Two participants indicated that during bedside nursing handoff that they “made corrections.” The ability to access and read provider notes [3, 39] and participate in multi-provider communications supports the ability of patients to verify content and catch errors [2, 19, 30]. Participants in this study identified that discussions about their care included unfamiliar jargon and often occurred only between practitioners without attention being given to the patient, even when they were alert and present. Research has shown that communication problems are inherent in the paternalistic provider structures still evident in patient care and these problems have the potential to leave patients feeling disenfranchised from their own care [27, 29]. Larger potential impacts on the healthcare system and patient include discouraging self-management of chronic conditions and reinforcing reliance upon providers.

Patients also identified interest in maintaining their own health histories and information but most had to “cobble together” a myriad of methods to keep track of their evolving condition during hospitalization. The participants in this study demonstrated great creativity at times in how they managed their own health information, such as taking pictures with a smart phone to get an electronic copy of a document. The lack of a consistent and user-friendly way for patients to participate and access their own healthcare information during hospitalization is an example of a care system that prioritizes provider concerns and discourages patient involvement [14]. Hospitals, in conjunction with patients and families, could work together to develop and share methods for managing patient health information which support the information needs of patients and providers [16, 32].

*Limitations.* Convenience and social media snowball sampling (SMSS) resulted in a highly activated and educated sample. Generalizability of the study findings is influenced by participant characteristics, including a predominately female sample. In addition, those interested in the topic, or who experienced issues with their hospitalization, may have been more likely to volunteer to participate. The PAM® instrument measures four developmental domains of patient activation: 1) believes active role is important, 2) confidence and knowledge to take action, 3) taking action, and 4) staying the course under stress [33]. The majority of our participants (91%) were level 3 or 4, indicating they were actively involved with self-management of their chronic disease and persistent in the face of obstacles. Future research is needed with a more diverse sample. In addition, our recruitment method resulted in four U.S. regions (Table 2) being represented but the southern region was over-represented. In addition, most hospitals were located in an urban (22) or suburban (10) setting with only one rural hospital represented.

## Conclusions

This study revealed that many hospitalized patients want to be included during provider discussions, such as nursing bedside handoff and medical rounds. While this very highly educated and highly activated sample expressed a desire to “know it all” further research is needed to investigate if there are differences in hospitalized patients desire for information based on individual factors, such as education or activation level. Future research is needed to explore how providers can accurately determine the readiness and desire for information on the individual patient level.

Participants identified provider behaviors that interfered with communication processes and led to concern about the completeness and accuracy of the shared information. Research is needed to learn more about provider perceptions, barriers, and facilitators to including patients in care discussions. Targeted interventions to improve how providers communicate with and care for patients – for example, around health literacy [1, 40, 41] – have been developed to address areas for improvement in the delivery of care. Such interventions could be used as a model for future educational efforts aimed at training providers to better consider, understand, and act on an understanding of patients’ health information needs.

## Additional file

Additional file 1: Benham-Hutchins Survey. Research team created survey questions. (PDF 293 kb)
