# Supplementary material for: “I want to know everything”: a qualitative study of perspectives from patients with chronic diseases on sharing health information during hospitalization
Source: BMC Health Serv Res. 2017 Aug 4;17:529. doi: 10.1186/s12913-017-2487-6 (PMC5544974; doi:10.1186/s12913-017-2487-6)
Supplement: Additional file 1: — Benham-Hutchins Survey. Research team created survey questions. (PDF 293 kb) [file 12913_2017_2487_MOESM1_ESM.pdf]

Q1 Consent to Participate in Internet Research (consent form). If you have reviewed the consent form and agree to participate in the study, please click "Next" to begin.

Eligibility Questions (Yes to q2-5 for inclusion)

Q2 Do you live in the United States?
- Yes (1)
- No (2)

Q3 Are you over 18?
- Yes (1)
- No (2)

Q4 Have you been diagnosed with a chronic disease (such as cancer, diabetes, or cystic fibrosis)?
- Yes (1)
- No (2)

Q5 Have you been hospitalized since receiving a diagnosis for your chronic disease?
- Yes (1)
- No (2)

Q6 What is your primary chronic disease? (select one)
- Arthritis (1)
- Cancer (2)
- Celiac Disease (3)
- Crohn's Disease (4)
- Cystic Fibrosis (5)
- Diabetes (Type 1) (6)
- Diabetes (Type 2) (7)
- Epilepsy (8)
- Heart Disease (9)
- Kidney Disease (10)
- Lupus (11)
- Other (12) ____________________

Q7 Do you have any additional or secondary chronic diseases? (select all that apply)
- Arthritis (1)
- Cancer (2)
- Celiac Disease (3)
- Crohn's Disease (4)
- Cystic Fibrosis (5)
- Diabetes (Type 1) (6)
- Diabetes (Type 2) (7)
- Epilepsy (8)
- Heart Disease (9)
- Kidney Disease (10)
- Lupus (11)
- Other (12) ____________________
- None (13)

Author Permission Required

Q8 What is your age?
Q9 Please select your gender.
- Female (1)
- Male (2)
- Prefer not to answer (4)

Q10 How do you describe yourself? (Please select the one option that best describes you.)
- American Indian/Alaskan Native (1)
- Asian (3)
- Black/African American (4)
- Caucasian/White (6)
- Mixed Race (7)
- Native Hawaiian/Pacific Islander (2)
- Other Race (those not listed) (5)

Q11 Are you Hispanic/Latino?
- Yes (1)
- No (2)

12 What is the highest grade or year of school you have completed?
- Never attended school or only attended kindergarten (1)
- Grades 1 through 8 (Elementary) (2)
- Grades 9 through 11 (Some high school) (3)
- Grade 12 or GED (High school graduate) (4)
- Some college or technical school (5)
- College graduate (6)
- Some Graduate School (8)
- Advanced degree (7)
- Other (9)

Q13 Date of First Chronic Disease Diagnosis
Q14 Date of First Cancer Diagnosis
Q15 What type(s) of cancer have you been diagnosed with? (list all)
Q16 Which type(s) of therapy have you received for your cancer? (select all that apply)
- Chemotherapy (1)
- Radiation (2)
- Surgery (3)
- Other (please specify) (4) ____________________

Q17 What is your current cancer status? (select one)
- Actively receiving treatment (1)
- In remission or no evidence of disease (2)
- Survivor (cancer-free for 5 or more years) (4)
- Other (please specify) (5) ____________________

Q18 Date of Hospital Admission (the one you will refer to for this survey)
Q19 How long were you in the hospital this time?
Q20 Why were you in the hospital this time?

Author Permisson Required

Author permission is required. Corresponding Author: Marge Benham-Hutchins, PhD, RN
mbenhamhutchins@austin.utexas.edu and margebhutch@yahoo.com

Q21 What type of setting was the hospital located in?
- o Urban (1)
- o Rural (2)
- o Suburban (3)
- o Other (4)

Q22 What state was it located in?

Q23 Managing your chronic disease involves keeping track of information across settings, treatments, and providers. Self-care refers to how you manage your own health care and is also called self-management. This includes activities such as taking medications, changing surgical bandages, knowing which symptoms to watch for, and coordinating the care you receive from multiple providers. First, we would like to know about how you currently manage your own health care.

Q24 Please share how you keep track of your health information as you go from provider to provider and setting to setting.

Q25 Please share how you keep track of your health information while you are in the hospital.

Q26 In your opinion, how well was your medical care organized while you were in the hospital?
- o Very Poorly Organized (1)
- o Poorly Organized (2)
- o Acceptable (3)
- o Well Organized (4)
- o Very Well Organized (5)

Q27 What examples come to mind, good or bad?

Q28 What kinds of medical information are you interested in knowing about when you are in the hospital?

Q29 What medical information do you prefer not to know about when you are in the hospital?

Q30 In your opinion, how often did your providers seem to know what they needed to know about you, your medical care, and what you needed?
- o Hardly Ever (7)
- o Usually Not (2)
- o Sometimes (3)
- o Usually (4)
- o Consistently (5)

Q31 What examples come to mind, good or bad?

Q32 Some hospitals offer their in-patients a "patient portal" -- a web page where you can look at your lab test results, make appointments, and send emails to your providers. While hospitalized, did you have access to your current health information through a patient portal?
- o Yes (1)
- o No (2)
- o I don't remember (3)

If Yes Is Selected, Then Skip To While in the hospital, how did you ac...If Yes Is Selected, Then Skip To What information was available throug...If No Is Selected, Then Skip To Some hospitals

Author Permission Required

provide a whiteboard o...If I don't remember Is Selected, Then Skip To Some hospitals provide a whiteboard o...

Q33 While in the hospital, how did you access the patient portal? (select all that apply)
- Personal Computer (1)
- Personal Tablet (2)
- Personal Phone (3)
- Hospital Provided Tablet (4)
- Hospital Provided Computer (5)
- Other (please specify) (6) ____________________

Q34 What information was available through the patient portal while you were in the hospital? (select all that apply)
- Test results and/or reports (1)
- Medications (2)
- Medical progress notes (3)
- New doctors' orders (4)
- Other (please specify) (5) ____________________

Q35 Some hospitals provide a whiteboard or bulletin board in patient rooms.

Q36 During your hospitalization, did the hospital display information about your care on a whiteboard or bulletin board in your room?
- Yes (1)
- No (2)
- I don't remember (3)

If Yes Is Selected, Then Skip To Did the whiteboard or bulletin board ...If No Is Selected, Then Skip To End of BlockIf I don't remember Is Selected, Then Skip To End of Block

Q37 Did the whiteboard or bulletin board display provider names?
- Yes (1)
- No (2)
- I don't remember (3)

If Yes Is Selected, Then Skip To Was the provider list kept up to date? If No Is Selected, Then Skip To Did the whiteboard display family mem...If I don't remember Is Selected, Then Skip To Did the whiteboard display family mem...

Q38 Was the provider list kept up to date?
- Hardly Ever (1)
- Usually Not (2)
- Sometimes (3)
- Usually (4)
- Consistently (5)

Author Permission Required

Author permission is required. Corresponding Author: Marge Benham-Hutchins, PhD, RN
mbenhamhutchins@austin.utexas.edu and margebhutch@yahoo.com

Q39 Did the whiteboard or bulletin board display family member contact information?
- Yes (1)
- No (2)
- I don't remember (3)

If Yes Is Selected, Then Skip To Was the family member contact informa...If No Is Selected, Then Skip To Did the whiteboard display your pain ...If I don't remember Is Selected, Then Skip To Did the whiteboard display your pain ...

Q40 Was the family member contact information kept up to date?
- Hardly Ever (1)
- Usually Not (2)
- Sometimes (3)
- Usually (4)
- Consistently (5)

Q41 Did the whiteboard display your pain medication schedule?
- Yes (1)
- No (2)
- I don't remember (3)
- I did not take pain medication (4)

If Yes Is Selected, Then Skip To Was information about your pain medic...If No Is Selected, Then Skip To Please list any other information dis...If I don't remember Is Selected, Then Skip To Please list any other information dis...If I did not take pain medication Is Selected, Then Skip To Please list any other information dis...

Q42 Was information about your pain medication schedule useful?
- Hardly Ever (1)
- Usually Not (2)
- Sometimes (3)
- Usually (4)
- Consistently (5)

Q43 Was the pain medication information kept up to date?
- Hardly Ever (1)
- Usually Not (2)
- Sometimes (3)
- Usually (4)
- Consistently (5)

Q44 Please list any other information displayed on the whiteboard or bulletin board.

Q45 When you are hospitalized, your providers exchange information about your care and discuss test results and treatment plans. These discussions often occur during medical team patient rounds and nursing change of shift report. Sometimes, these activities occur in the

Author Permission Required

patient's room. We have some questions about your participation in these activities if they occurred in your hospital room.

Q46 During your hospitalization, did the nurses give a change of shift report in your hospital room?
- Yes (1)
- No (2)
- I don't remember (3)

If Yes Is Selected, Then Skip To How often did nursing change of ...If No Is Selected, Then Skip To End of BlockIf I don't remember Is Selected, Then Skip To End of Block

Q47 How often did nursing change of shift report occur in your hospital room?
- Hardly Ever (3)
- Usually Not (4)
- Sometimes (5)
- Usually (6)
- Consistently (7)
- I don't remember (8)

Q48 Did the nurses explain what they were doing?
- Hardly Ever (3)
- Usually Not (4)
- Sometimes (5)
- Usually (6)
- Consistently (7)
- I don't remember (8)

Q49 Were you invited to participate?
- Hardly Ever (2)
- Usually Not (3)
- Sometimes (4)
- Usually (5)
- Consistently (6)
- I don't remember (7)

Q50 What did you do during the process? Select all that apply.
- Listen (1)
- Ask questions (2)
- Answer questions (3)
- Other (4) ____________________
- I don't remember (5)

Q51 What factors do you think encourage patient participation in bedside nursing shift report?
Q52 What factors do you think discourage patient participation in bedside nursing shift report?

Author Permission Required

Q53 Your providers sometimes meet as a group to discuss your results and treatment plans. This is called medical rounds and commonly includes your doctor, residents, interns, and nurses.During your hospitalization, did your doctors and other providers discuss your medical care as a group (medical rounds) in your hospital room?

- ○ Yes (1)
- ○ No (2)
- ○ I don't remember (3)

If Yes Is Selected, Then Skip To How often did medical rounds occur in...If No Is Selected, Then Skip To End of BlockIf I don't remember Is Selected, Then Skip To End of Block

Q54 How often did medical rounds occur in your hospital room?

- ○ Hardly Ever (1)
- ○ Usually Not (2)
- ○ Sometimes (3)
- ○ Usually (4)
- ○ Consistently (5)
- ○ I don't remember (6)

Q55 Did the medical team explain what they were doing?

- ○ Hardly Ever (1)
- ○ Usually Not (2)
- ○ Sometimes (3)
- ○ Usually (4)
- ○ Consistently (5)
- ○ I don't remember (6)

Q56 Were you invited to participate?

- ○ Hardly Ever (1)
- ○ Usually Not (2)
- ○ Sometimes (3)
- ○ Usually (4)
- ○ Consistently (5)
- ○ I don't remember (6)

Q57 What did you do during the process? Select all that apply.

- ○ Listen (1)
- ○ Ask questions (2)
- ○ Answer questions (3)
- ○ Other (please specify) (4) ____________________
- ○ I don't remember (5)

Q58 What factors do you think encourage patient participation in medical rounds?
Q59 What factors do you think discourage patient participation in medical rounds?

Author Permission Required

Author permission is required. Corresponding Author: Marge Benham-Hutchins, PhD, RN
mbenhamhutchins@austin.utexas.edu and margebhutch@yahoo.com

Q60 We would like to know whether, when you were discharged, you felt prepared to take care of yourself at home.

Q61 When you first got home from the hospital, how confident were you that you knew what you would need to do to take care of your medical needs?
- Very Uncertain (1)
- Uncertain (2)
- Neutral (3)
- Confident (4)
- Very Confident (5)

Q62 When you first got home from the hospital, how confident were you that you had the things you would need, such as equipment and prescriptions?
- Very Uncertain (1)
- Uncertain (2)
- Neutral (3)
- Confident (4)
- Very Confident (5)

Q63 When you first got home from the hospital, how confident were you that you knew what to watch out for to recognize problems?
- Very Uncertain (1)
- Uncertain (2)
- Neutral (3)
- Confident (4)
- Very Confident (5)

Q64 When you first got home from the hospital, how confident were you that you knew what provider to contact if you had questions or a problem?
- Very Uncertain (1)
- Uncertain (2)
- Neutral (3)
- Confident (4)
- Very Confident (5)

Author Permission Required

Q65 We would like to know whether, as time went by (a week or more after discharge), you felt prepared to take care of yourself at home.

Q66 As time went by, did you find that you actually did know what you needed to do to take care of yourself?
- Yes (1)
- No (2)
- I don't remember (3)

If I don't remember Is Selected, Then Skip To As time went by, did you find that yo...

Q67 What examples come to mind, good or bad?

Q68 As time went by, did you find that you had the things you needed -- equipment, prescriptions, or anything else?

- Yes (1)
- No (2)
- Not applicable (4)
- I don't remember (3)

If I don't remember Is Selected, Then Skip To Did you find that you knew what to wa...If Not applicable Is Selected, Then Skip To Did you find that you knew what to wa...

Q69 What examples come to mind, good or bad?

Q70 As time went by, did you find that you did know what to do if problems developed?

- Yes (1)
- No (2)
- Not applicable (4)
- I don't remember (3)

If I don't remember Is Selected, Then Skip To Did you find that you knew whom to co...If Not applicable Is Selected, Then Skip To Did you find that you knew whom to co...

Q71 What examples come to mind, good or bad?

Q72 As time went by, did you find that you knew whom to contact if you had questions or a problem?

- Yes (1)
- No (2)
- Not applicable (4)
- I don't remember (3)

If I don't remember Is Selected, Then Skip To Did you find that you got the help yo...If Not applicable Is Selected, Then Skip To Did you find that you got the help yo...

Q73 What examples come to mind, good or bad?

Q74 Did you find that you got the help you needed, when you needed it?

- Yes (1)
- No (2)
- Not applicable (4)
- I don't remember (3)

If I don't remember Is Selected, Then Skip To End of BlockIf Not applicable Is Selected, Then Skip To End of Block

Q75 What examples come to mind, good or bad?

Author Permission Required

Q76 The following questions contain various statements that people make when they talk about their health. Please indicate how much you agree or disagree with each statement as it applies to you personally; if a statement does not apply to you, select N/A. Keep in mind that your answers should reflect your opinions and not what you think the doctor wants you to say.
Q77 – Q86 Patient Activation Measure Questions [Information on licensing the PAM® survey is available through Insignia Health (http://www.insigniahealth.com/products/pam-survey).]

Q87 Thinking back on your hospitalization, is there anything else that you think could be done during hospitalization to help prepare you to resume self-care after you went home?

Q88 You have completed the survey. Please click "Next" to submit your answers.

Author Permission Required
